# Azole Resistance in *Aspergillus* Species Isolated from Clinical Samples: A Nine-Year Single-Center Study in Turkey (2015–2023)

**DOI:** 10.3390/jof11090659

**Published:** 2025-09-07

**Authors:** Zeynep Yazgan, Reyhan Çalışkan, Gökhan Aygün

**Affiliations:** 1 Department of Medical Microbiology, Cerrahpasa School of Medicine, Istanbul University-Cerrahpasa, Istanbul 34320, Turkey; gokhanaygun67@yahoo.com; 2 Department of Medical Microbiology, Basic Medical Sciences, Faculty of Medicine, Samsun University, Samsun 55030, Turkey; reyhan.caliskan@samsun.edu.tr; 3 Department of Infectious Diseases and Clinical Microbiology, Cerrahpasa School of Medicine, Istanbul University-Cerrahpasa, Istanbul 34320, Turkey

**Keywords:** *Aspergillus*, azole resistance, Turkey, prevalence, antifungal susceptibility

## Abstract

Azole-resistant mycotic infections pose an escalating global health threat, with an estimated 6.5 million invasive fungal infections (IFIs) annually leading to 3.8 million deaths, 68% directly caused by IFIs. While azole antifungals are the cornerstone of treatment, emerging resistance, mainly due to gene mutations and efflux pump overexpression, is a major concern. This study, spanning 2015–2023, investigated azole resistance in clinical *Aspergillus* isolates in Turkey, a region lacking comprehensive surveillance. Of 200 causative isolates, *A. fumigatus* accounted for 45% and respiratory samples 57%. Overall azole resistance was 7%, rising to 11% for *A. fumigatus*. Findings highlight the persistent challenge of azole resistance, emphasizing the critical need for continued local and global surveillance to inform treatment guidelines and public health interventions. Despite limitations, including a single-center focus, this research provides crucial epidemiological insights into the evolving landscape of antifungal resistance in Turkey.

## 1. Introduction

Mycotic infections constitute an escalating global health threat, presenting significant challenges to public health systems worldwide due to resistance [1]. Updated estimations indicate a yearly incidence of 6.5 million invasive fungal infections (IFIs), which lead to 3.8 million deaths. Of these fatalities, approximately 2.5 million (68%, with a reported range of 35–90%) are directly caused by IFIs [2]. Aspergillosis represents one of the most significant opportunistic fungal infections, particularly affecting immunocompromised patients including those with hematological malignancies, solid-organ transplant recipients, patients receiving broad-spectrum antibiotics, and individuals in ICU [3,4,5]. *Aspergillus fumigatus* remains the predominant causative agent, accounting for approximately 56% of invasive aspergillosis cases; other species within the *Aspergillus* complex, such as *A. flavus*, *A. niger*, and *A. terreus*, follow [6]. The clinical spectrum ranges from allergic manifestations to life-threatening invasive diseases, with mortality rates exceeding 50% despite optimal therapy [7,8,9].

Azole antifungals, particularly itraconazole, voriconazole, and posaconazole, constitute the cornerstone of aspergillosis treatment and prophylaxis [10,11]. These agents target the fungal cytochrome P450 enzyme lanosterol 14α-demethylase (Cyp51A), disrupting ergosterol biosynthesis and compromising cell membrane integrity [12]. However, the emergence and increasing prevalence of azole resistance in *Aspergillus* species has become a major clinical concern, significantly impacting therapeutic outcomes and patient prognosis [13].

Azole resistance in *Aspergillus* is mainly caused by mutations in the cyp51A gene that reduce azole binding or increase gene expression. Overexpression of efflux pump genes lowers intracellular drug levels. Additional mechanisms include mutations, activation of stress response pathways as well as biofilm formation and exogenous cholesterol uptake, all contributing to reduced azole susceptibility [14]. The widespread use of azoles in both clinical settings and agriculture, particularly demethylation inhibitor (DMI) fungicides, has significantly contributed to the environmental selection and proliferation of resistant *Aspergillus* strains [15,16,17].

Global surveillance efforts have revealed substantial geographical variations in azole resistance rates, ranging from less than 1% to around 80% in some regions [18]. Despite the implementation of various monitoring initiatives globally, comprehensive epidemiological data on azole resistance remain limited across many regions. This data scarcity is particularly pronounced in Turkey, where systematic surveillance of azole-resistant *Aspergillus* species is notably insufficient. The existing Turkish studies are characterized by limited temporal scope and a lack of recent data on resistance prevalence rates across different investigations [19,20]. This gap in knowledge is critical, as local epidemiological insights are essential for informing clinical treatment guidelines, implementing effective public health interventions, and understanding the regional dynamics of anti-fungal resistance. Therefore, this study aims to determine the prevalence of azole resistance in clinical *Aspergillus* isolates in Turkey.

## 2. Methods

### 2.1. Clinical Isolates

The isolates identified as *Aspergillus* spp. from various clinical specimens (abscess, corneal abscess, biopsy, tissue and respiratory specimens) between 2015 and 2023 in the Medical Microbiology Mycology Laboratory of Cerrahpaşa Medical Faculty Hospital were evaluated. The isolates were further analyzed with a total of 200 *Aspergillus* isolates stored at −80 °C. The study was approved by the Istanbul University-Cerrahpaşa Rectorate Clinical Research Ethics Committee (decision number: 2024-992771; Annex 1).

For species level identification, the isolates were inoculated on Sabouraud dextrose agar (SDA; HiMedia (Mumbai, India)) and potato dextrose agar (PDA; Difco, Franklin Lakes, NJ, USA) and incubated at 25 °C and 35 °C. All colonies that were morphologically similar to *Aspergillus* spp. were isolated as pure cultures. Macroscopic and microscopic morphological features and thermotolerance test (growth at 45 °C) were evaluated for the identification of *Aspergillus* spp. isolates [21,22]. Furthermore, *Aspergillus* isolates exhibiting azole resistance were identified to the species level by MALDI-TOF MS (MALDI Biotyper, Bruker Daltonik GmbH, Bremen, Germany) in accordance with the method recommended for filamentous molds. Additionally, molecular DNA sequencing utilising the ITS1-F (5′-TCCGTAGGTGAACCTGCGG-3′) and ITS4-R (5′-TCCTCCGCTTATTGATATATGC-3′) primers (Sentromer Miami, USA DNA Technologies LLC, Miami, USA) was employed [23].

In the study group, the distinction between causative agent or colonizer was based on positive direct and stained microscopic examination and cultures (or culture growth of consecutive samples), serum or BAL (bronchoalveolar lavage) galactomannan positivity, radiological findings, histopathology results and clinician opinion [24,25]. Isolates that fell outside these criteria and were evaluated in favour of contamination were excluded from the study. In cases with multiple growths, the first isolate was further analyzed.

### 2.2. Antifungal Susceptibility Tests

RPMI agar was used as medium for susceptibility tests. In all isolates, antifungal susceptibility tests were performed by the broth microdilution method to confirm antifungal susceptibility to azole group antifungals in samples in which resistance was determined by gradient test and/or agar plate method. The European Committee on Antimicrobial Susceptibility Testing (EUCAST E.Def 7.3, E.Def 9.4 and E.Def 11.0; Version 3.0, valid from 18 January 2022) guidelines updated in 2022 were used to evaluate antifungal susceptibility tests [26]. The epidemiological thresholds and clinical cut-off values were taken into consideration.

**Gradient Test (E-test):** *Aspergillus* spp. were spread on RPMI 1640 (Sigma Chemical Co, St Louis, Mo, USA) media (with 2% glucose) with the aid of sterile swabs. Following vortexing of the suspension, E-test strips (ITZ, VOR, POS; Biomerieux Lyon, France) were placed on the medium with the assistance of forceps. The medium was then evaluated following incubation at 37 °C for 24–48 h. Antifungal susceptibility tests using the gradient test method were evaluated at 24 and 48 h for *A. fumigatus* complex, *A. flavus* and *A. niger*, at 48 h for *Aspergillus* spp. and *A. terreus*, and at 72 h for some rare *Aspergillus* spp. species with slow growth. If any azole MIC value was found at or above the resistance limit value, the antifungal susceptibility test was repeated for confirmation.

**Agar screening plate method:** Itraconazole (4 mg/L), voriconazole (2 mg/L) and posaconazole (0.5 mg/L) were added to RPMI 1640 (2% glucose) medium, which had been prepared according to EUCAST E.DEF 10.1 recommendations and dispensed into sterile 35 mm × 10 mm diameter (~4–5 mL) Petri dishes (Greiner, Germany). Antifungal-free media was also prepared and utilised as control plates. Growths on screening plates containing RPMI 1640 (2% glucose) agar with voriconazole, itraconazole and posaconazole added and without antifungal drugs (positive control) and on control plates were evaluated.

**Microdilution method:** Antifungal susceptibility tests were prepared and performed using the CLSI M38-A2 reference microdilution method. Antifungal susceptibility tests were performed by broth microdilution method to confirm the antifungal susceptibility of the isolates that were resistant to azole group antifungals by gradient test and agar plate method.

EUCAST (European Committee on Antimicrobial Susceptibility Testing) has updated the minimum inhibitory concentration (MIC) limit values for *A. fumigatus* as of 2023–2024 [27]. EUCAST MIC limit values of azole group antifungals for *A. fumigatus*:

1. Itraconazole: Susceptible (S): ≤1 mg/L; Resistant (R): >1 mg/L;
2. Voriconazole: Susceptible (S): ≤1 mg/L; Resistant (R): >1 mg/L;
3. Posaconazole: Susceptible (S): ≤0.25 mg/L; Resistant (R): >0.25 mg/L.

Statistical analysis

Statistical analysis was performed to support comparisons within the dataset (IBM SPSS Statistics 30). A Chi-square test for trend was used to evaluate whether there was a significant change in the proportion of azole-resistant isolates over the nine-year study period. To compare the prevalence of azole resistance between *Aspergillus* fumigatus and all other *Aspergillus* species combined, a Chi-square test of independence was employed. For all statistical tests, a *p*-value of <0.05 was considered to indicate statistical significance.

## 3. Results

*Aspergillus* isolation was detected in a total of 471 clinical specimens from various clinical samples in the Medical Microbiology Mycology Laboratory of Cerrahpaşa Medical Faculty Hospital. Of these samples, 200 were considered to be ‘causative’. 271/471 of our isolates were identified as colonizers, whereas 200/471 were identified as causative agents.

### 3.1. Distribution of Isolates

**Figure 1:**
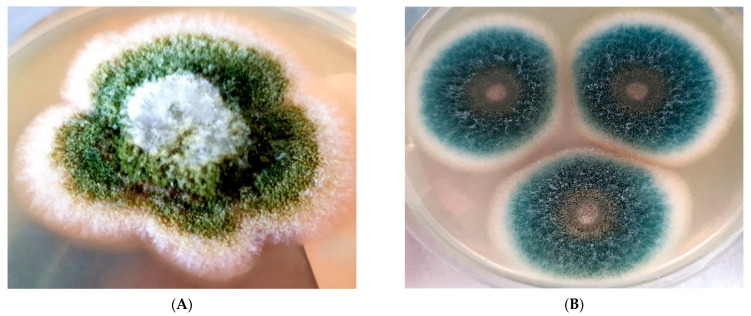

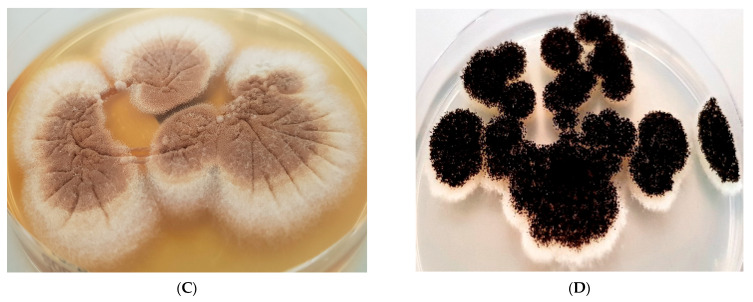
Colony morphology of different *Aspergillus* species on Sabouraud Dextrose Agar. ((**A**): *A. flavus*, (**B**): *A. fumigatus*, (**C**): *A. terreus*, (**D**): *A. niger*).

A total of 200 *Aspergillus* species were included in the study, including 90 *A. fumigatus* complex (45%), 46 *A. flavus* (23%), 10 *A. terreus* (5%), 9 *A. niger* (4.5%) and 45 *Aspergillus* spp. (22.5%). The distribution of isolates according to samples is shown in Table 1. Colony morphology of different *Aspergillus* species on Sabouraud Dextrose Agar (A: *A. flavus*, B: *A. fumigatus*, C: *A. terreus*, D: *A. niger*) in Figure 1.

In *A. fumigatus*, 60% of the clinical samples (54/90) studied belonged to respiratory samples. In A. flavus, the proportion of respiratory samples was 54% (25/46). In *Aspergillus* spp., the proportion of respiratory samples was 47% (21/45). Among all clinical specimens identified as *Aspergillus*, the proportion of respiratory specimens (114/200) was 57%.

### 3.2. Antifungal Susceptibility Patterns

**Figure 2:**
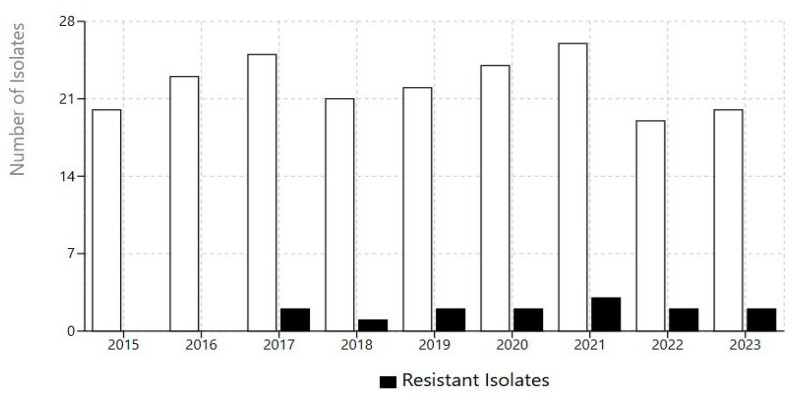
Annual distribution and antimicrobial resistance patterns of *Aspergillus* isolates.

In the study covering a 9-year period between 2015 and 2023 in both *Aspergillus* spp. and *A. fumigatus* species, the rate of azole resistance was 14/200 (7%) in all *Aspergillus* isolates according to the gradient test method. This rate for *A. fumigatus* was 10/90 (11%). Also, year and resistance *Aspergillus isolates* number showed in Figure 2.

The distribution of resistant isolates according to the gradient test results of our study; 10 isolates belong to *Aspergillus fumigatus* complex, 1 isolate to *Aspergillus terreus*, 1 isolate to *Aspergillus niger* and 2 isolates to *Aspergillus pseudoglaucus*.

A Chi-square test of independence revealed that the rate of azole resistance was significantly higher in A. fumigatus compared to other *Aspergillus* species (*p* < 0.05). While the yearly percentage of resistant isolates fluctuated, a Chi-square test for trend revealed no statistically significant increase in resistance rates over the nine-year study period (*p* > 0.05).

According to the antifungal susceptibility results of the azole-resistant isolates determined by gradient test, agar plate and microdilution methods, those with an ITR MIC value of 4 µg/mL by gradient test and microdilution methods produced positive results. Agar plate method; VOR MIC value of 2 µg/mL and POS MIC value of 0.5 µg/mL gave positive results. According to the agar plate method, five patients showed resistance in all cases.

According to EUCAST (European Committee on Antimicrobial Susceptibility Testing), MIC (minimum inhibitory concentration) limit values for *A. fumigatus* complex updated as of 2023–2024; when the E-test results are evaluated; the MIC value of one isolate in VOR is >32. In ITR, 10 isolates had MIC values greater than 1 µg/mL. In ITR, 13 isolates had MIC values equal to 1 µg/mL. Two isolates had MIC values greater than 0.25 µg/mL in the POS. One *A. fumigatus* was found to be pan-azole resistant. For *A. fumigatus* isolates, no isolates showing mono-azole resistance to POS were found. Table 2 shows antifungal susceptibility test results of *Aspergillus fumigatus* complex E-test.

According to the E-test results for *Aspergillus flavus*, there were three isolates with a MIC of 1 µg/mL in ITR (6%). No isolates were found at ECOFF MIC values that were determined in VOR and POS. Table 3 shows antifungal susceptibility test results of *Aspergillus flavus* E-test.

According to the *Aspergillus terreus* E-test results, no isolate was found at ECOFF MIC values determined in VOR and POS. VOR and ITR MIC of 1 µg/mL were detected in one *A. terreus* isolate. According to *Aspergillus niger* E-test results; no isolate was found at ECOFF MIC values determined in VOR, ITR and POS. The ITR MIC value of an *A. niger* isolate was 2 µg/mL One *Aspergillus pseudoglaucus* isolate had a high MIC value in the gradient test, and another *A. pseudoglaucus* isolate had a high MIC value in the gradient test.

## 4. Discussion

The major findings of this study indicate that *A. fumigatus* is the most common species (45%) and that respiratory samples are the predominant source (57%). Overall azole resistance was 7%, increasing to 11% for *A. fumigatus* during our nine-year surveillance in Turkey. The first azole-resistant isolate was identified in 2017. When the distribution of resistance rates among *Aspergillus* spp. isolates was analyzed by year, no significant differences were detected. The two pan-azole resistant isolates were identified in 2017 and 2019. The isolate with VOR and POS resistance was from 2020. Two isolates exhibiting ITR resistance and one isolate showing VOR resistance were identified in 2021. Although the overall increase in MIC values and the prevalence of resistant *Aspergillus* spp. did not show a sharp rise over the years.

The ongoing emergence of resistance mechanisms continues to compromise the efficacy of azoles in treating aspergillosis, directly affecting patient outcomes [6]. Despite this growing concern, comprehensive epidemiological data on azole resistance in *Aspergillus fumigatus* in Turkey remain limited. Previous multi-center research in Turkey reported an overall azole resistance rate of 3.3% in *A. fumigatus* isolates. This resistance was predominantly observed in three centers characterized by low isolation rates of the pathogen [19]. Furthermore, an earlier three-year surveillance study identified azole resistance in 10.2% of *A. fumigatus* isolates, with the TR34/L98H mutation accounting for the majority (86.8%) of these cases [20]. The different rates in reported resistance rates across various Turkish studies (e.g., this study’s 11% compared to 3.3% and 10.2%) suggest potential epidemiological shifts over time, methodological variations between studies or a combination of these factors. Several factors may drive azole resistance in Turkey: extensive agricultural DMI fungicide use selecting for resistant A. fumigatus, prolonged azole therapy in patients with chronic pulmonary diseases, urban construction facilitating spore dispersal, and diverse climatic conditions creating regional variation [19]. Limited routine susceptibility testing likely results in under-detection of resistant strains. These studies, while valuable, represent data collected over short intervals; the latter study, for instance, was conducted over five years ago. This temporal limitation suggests potential for regional variations in resistance rates or evolving epidemiological trends that may not be captured by the existing literature.

In various studies conducted with *A. fumigatus* isolates, the highest resistance rate was observed to ITZ. Itraconazole (ITZ) resistance in *Aspergillus* species, an azole that is a crucial agent in aspergillosis management and prophylaxis, has been globally investigated, with reported rates ranging from 1% to 27% [28,29]. In Europe, azole resistance typically ranges from 1–5%, though it can reach up to 10% in countries like the Netherlands, a phenomenon largely attributable to agricultural fungicide use.

In the study by Mortensen et al. (2011), all patients with *A. fumigatus* strains exhibiting reduced susceptibility had received azole antifungals [30]. Burgel et al. (2012) reported the presence of isolates with reduced susceptibility in 1.2% of patients who had not received ITZ treatment [31]. Morio et al. (2012) found that azole resistance was associated with prior antifungal treatment in their patient cohort [32].

A higher prevalence of azole-resistant isolates was found in some patients with specific risk factors, such as prior long-term azole exposure. The resistance rate of isolates obtained from patients with chronic pulmonary aspergillosis has been reported to be up to 59% [33]. The emergence of resistance is partly due to inappropriate antifungal use [34]. The ITZ resistance rate was reported as 14% in a total of 929 *A. fumigatus* isolates obtained from patients with cystic fibrosis between 2015 and 2019 [35].

Secondary azole resistance in *Aspergillus* isolates poses a significant clinical threat, potentially exacerbated by agricultural fungicide use. This cross-resistance between clinical and environmental settings may compromise both therapeutic efficacy and agricultural disease control [36]. In our study, patient epidemiological information and antifungal treatment history were not collected, thus precluding the determination of the possible route of resistance acquisition.

Studies indicate a growing concern regarding azole resistance in *A. fumigatus*. Fischer et al. (2014) identified azole resistance in 1.14% of *A. fumigatus* isolates from respiratory samples, which they attributed to mutations in the cyp51A gene [37]. Prigitano et al. (2021) reported higher azole resistance rates of 4.9% for *Aspergillus* species and 6.6% for *A. fumigatus sensu stricto* [38]. The prevalence of azole-resistant *A. fumigatus* has increased significantly, particularly in European countries, with global resistance rates varying widely from 0.55–30% across different geographical regions [39,40,41,42,43,44,45].

To adequately describe the extent of the threat posed by azole-resistant environmental *A. fumigatus* in Southeast Asia, a study in Vietnam investigated antifungal susceptibilities and environmental azole concentrations in soil. Of the 119 isolates, 55% were resistant to ITZ, 65% to POS, and 50% to VOR. cyp51A gene sequence analysis revealed that 38 of 56 resistant *A. fumigatus* isolates carried known resistance mutations, most frequently TR34/L98H (34/38) [46].

The ITZ resistance rates of *A. fumigatus*, *A. flavus*, *A. terreus*, and *A. niger* from Asia and the Western Pacific Region were reported as 2.1%, 0%, 0%, and 2.2%, respectively [28]. Europe also exhibited the highest NWT rates for ITZ (1.7%) compared to other regions (0.0–0.7%) [29]. Pfaller et al. (2024) investigated trends (2017–2021) in the activity of mold-acting azole agents against *A. fumigatus* clinical isolates from Europe and North America [47]. In their study, 14 different cyp51 alterations were detected in 44 of 79 non-wild type (NWT) isolates. In our study, the resistance rate in *A. fumigatus* isolates (n = 90) was 11%, based on an ITZ MIC resistance breakpoint of >1 mg/L. In a study involving seven centers in the Netherlands, Van der Linden et al. (2011) screened 2062 clinical *Aspergillus* isolates (comprising different species) for ITZ resistance between 2007 and 2009, reporting a prevalence of 5.3% ITZ resistance among the 1792 A. fumigatus isolates [48]. Subsequently, from 2941 *A. fumigatus* isolates, 47 resistant isolates were identified [49]. Of these, the TR34/L98H mutation was present in 48.9% (n = 23) and TR46/Y121F/T289A in 6.3% (n = 3). In Belgium and the Netherlands, the TR46/Y121F/T289A mutation was first detected in panazole-resistant strains in 2014 [50,51]. In our study, 14 resistant species were identified, and the most likely resistance mechanism involves the multifactorial nature of azole resistance in *Aspergillus* fumigatus. This resistance is primarily attributed to mutations in the target cyp51A gene, sometimes in combination with synergistic hmg1 mutations. Additionally, resistance can be mediated by the overexpression of drug efflux pumps, which may be driven by upregulated energy metabolism [52].

Studies investigating azole resistance in *Aspergillus* species have often been limited to isolates that could be cultivated in a laboratory setting. The application of molecular methods for the detection of *Aspergillus* species enhances the precision with which true rates can be determined [53]. To validate the resistance of isolates to azole-group antifungals, which was initially identified using the gradient test and agar plate method, antifungal susceptibility testing was subsequently performed with the broth microdilution method. In a study comparing the E-test and reference microdilution methods for determining VOR and ITZ MICs for 376 *Aspergillus* spp. isolates, Pfaller et al. (2003) reported essential agreement with liquid microdilution rates of 98% for VOR and 96% for ITZ [54]. Using the gradient test, Delliere et al. (2020) demonstrated a 91.7% detection rate for azole-resistant strains based on ITZ MICs [55]. Consequently, the gradient assay was identified as a valuable method for screening azole-resistant *A. fumigatus* clinical isolates. In a comparative evaluation of different gradient diffusion tests for the detection of azole resistance in *A. fumigatus*, one such test, the E-test, was evaluated for ITR, POS, and VOR against the EUCAST liquid medium microdilution method. The performance of both assays was satisfactory in general; however, the POS MIC in the gradient assay demonstrated suboptimal agreement with the liquid medium microdilution, owing to an overestimation of the MIC. Essential agreement between the gradient diffusion strips (from two different manufacturers) and liquid microdilution for 24 *A. fumigatus* isolates, including those with known resistance mechanisms, ranged from 83% to 100%, with no significant error. Categorical agreement using EUCAST limit values was 96–100% for ITR and VOR, whereas for POS, it was only 33% and 83% for the two different manufacturers [56].

Lamoth and Alexander (2015) compared 154 *Aspergillus* spp. isolates using E-test and broth microdilution as antifungal susceptibility tests [57]. They reported that basic agreement between the two methods, defined as within ±2 dilutions and ≥90%, was considered acceptable. Within this study, the basic agreement for VOR was >90% for the most potentially susceptible species. However, E-test MIC values for POS were consistently lower for *Aspergillus* spp., demonstrating a basic agreement of less than 90%. Therefore, they concluded that additional work on molecularly characterized triazole-resistant *Aspergillus* isolates is required to confirm the ability of the E-test method to detect VOR and POS resistance among *Aspergillus* spp. [57]. Conversely, the majority of *A. fumigatus* isolates in the study by Tan et al. (2024) exhibited low MIC values to POS compared to other tested antifungals [58].

Due to the prevalence of aspergillosis and the associated spectrum of fungi, infection sites, age, climate, geographical conditions, agricultural activities, and other factors, worldwide resistance rates vary widely from country to country and even from region to region within a country. It is important to examine data in detail on current and global azole resistance rates and epidemiological reports for a specific country or region. The presence and frequency of secondary azole resistance in *A. fumigatus* in many countries is yet to be fully determined. The *Aspergillus* Resistance Surveillance Working Group (ISHAM/ECMM) has emphasized the importance of facilitating surveillance studies to determine the epidemiology of resistance in countries where data are currently lacking and to provide greater insight into clinical outcomes [59].

A limitation of this study is its conduct in a single center and its exclusive focus on clinical isolates. Furthermore, broth microdilution (BMD) susceptibility tests were not performed on all isolates. Isavuconazole susceptibility was not tested due to unavailability during the study period, which we acknowledge as a limitation. Since this study focused primarily on the frequency and mechanisms of resistance, utilizing retrospectively detected isolates, comprehensive clinician opinion and patient epidemiological information, while collected for agent-isolate differentiation, were not sufficient for in-depth clinical evaluation and thus were not included in the primary analysis.

These results highlight the need for robust antifungal stewardship in Turkey. Routine susceptibility testing of clinically relevant *Aspergillus* isolates, particularly in tertiary centers, should be implemented. Education on optimal antifungal prescribing and de-escalation is essential to minimize unnecessary azole exposure. Coordinated efforts between health authorities and the agricultural sector are required to regulate azole fungicide use and limit environmental selection pressure. A national surveillance network integrating clinical and environmental data would facilitate early detection of resistance and inform region-specific treatment guidelines.

## 5. Conclusions

This study, spanning 2015–2023, reveals critical insights into azole resistance in clinical *Aspergillus* isolates in Turkey. *A. fumigatus* was the dominant species (45%), primarily from respiratory samples (57%). Overall azole resistance was 7%, rising to 11% for *A. fumigatus*, including pan-azole resistant strains. Itraconazole resistance rates in *A. fumigatus* mirrored global trends. Despite limitations (single-center, clinical isolates), this research highlights the persistent threat of azole resistance, impacting aspergillosis treatment.

## Figures and Tables

**Table 1:** Distribution of isolates according to samples.

Clinical Isolates	TISSUE		ABSCESS		RESPIRATORY			OTHER				TOTAL
	Tissue	Abscess	Cornea Abscess	BAL	Sputum	ETA	Nasal Sinus Content	CSF	Joint Fluid	Catheter	Pleura	
*A. fumigatus*	11	16	1	24	20	10	—	2	2	3	1	**90**
*A. flavus*	13	3	4	12	10	1	2	—	—	—	1	**46**
*Aspergillus* spp.	11	8	3	9	10	2	—	—	—	—	—	**43**
*A. pseudoglaucus*	1	—	1	—	—	—	—	—	—	—	—	**2**
*A. terreus*	—	2	—	3	4	—	—	—	1	—	—	**10**
*A. niger*	2	—	—	4	3	—	—	—	—	—	—	**9**

**Abbreviations:** BAL: Bronchoalveolar lavage, ETA: Endotracheal aspiration, CSF: Cerebrospinal fluid, —: No isolates found.

**Table 2:** Antifungal Susceptibility Test Results: *Aspergillus* fumigatus complex E-test, MIC µg/mL (n = 90).

Anti Fungal	No (n=)	≤0.002	0.003	0.004	0.008	0.012	0.016	0.023	0.032	0.047	0.064	0.094	0.125	0.19	0.25	0.38	0.50	0.75	1.0	1.5	4	>32
**VOR**	**88**	—	—	—	—	—	—	—	2	—	1	6	23	17	32	4	2	—	—	—	—	1
**ITR**	**84**	—	—	—	—	—	—	—	—	—	—	—	—	4	5	24	16	12	13	9	1	—
**POS**	**87**	7	6	4	2	4	1	2	3	11	5	13	13	10	4	1	—	1	—	—	—	—

**Abbreviations: VOR:** Voriconazole, **ITR:** Itraconazole, **POS:** Posaconazole, **—:** No isolates at this concentration.

**Table 3:** Antifungal Susceptibility Test Results: *Aspergillus* flavus E-test, MIC µg/mL (n = 46).

Anti Fungal	No (n=)	≤0.002	0.012	0.016	0.023	0.032	0.047	0.064	0.094	0.125	0.19	0.25	0.38	0.50	0.75	1.0
**VOR**	**46**	—	—	—	—	—	2	4	6	9	9	6	7	2	1	—
**ITR**	**46**	—	—	—	—	—	—	1	—	3	5	5	15	12	2	3
**POS**	**46**	4	1	3	2	4	7	9	5	7	4	—	—	—	—	—

**Abbreviations: VOR:** Voriconazole, **ITR:** Itraconazole, **POS:** Posaconazole, **—:** No isolates at this concentration.

## Data Availability

The original contributions presented in this study are included in the article. Further inquiries can be directed to the corresponding author.
